# The complete mitochondrial genome of *Monolepta hieroglyphica* (Motschulsky) (Coleoptera: Chrysomelidae)

**DOI:** 10.1080/23802359.2021.1951138

**Published:** 2021-07-15

**Authors:** Qi He, Xianmei Song, Hongwen Ma, Yanbo Yin

**Affiliations:** a Institution of Crop Research, Ningxia Academy of Agricultural and Forestry Science, Yinchuan, People’s Republic of China; b School of Agriculture, Ningxia University, Yinchuan, People’s Republic of China

**Keywords:** *Monolepta hieroglyphica* (Motschulsky), mitochondrial genome, phylogeny

## Abstract

*Monolepta hieroglyphica* (Motschulsky) (Coleoptera: Chrysomelidae) is an important agricultural insect pest. In this study, the complete mitochondrial genome of *M. hieroglyphica* was sequenced using Illumina HiSeq X Ten. The mitogenome was 16,213 bp long and comprised 13 protein-coding genes (PCGs), two ribosomal RNA genes (rRNAs), 22 transfer RNA genes (tRNAs) and a putative control region (CR). The nucleotide composition of the *M. hieroglyphica* mitochondrial genome was significantly biased (A, G, C and T accounted for 41.04%, 8.01%, 11.76% and 39.18%, respectively) with 80.23% A + T content. Two rRNAs were located between tRNA-Leu and the CR, separated by tRNA-Val. The CR, located between 12 s rRNA and tRNA-Ile, was 1661 bp long. The length of the 22 tRNAs ranged from 61 to 71 bp. Phylogenetic analyses of 29 Chrysomelidae-Galerucinae species based on 13 mitochondrial protein-coding genes reconstructed using Bayesian 3.2.0 showed that the *M. hieroglyphica* mitogenome was clustered with the existing three different species of the *Monolepta* genus mitogenomes in a monophyletic manner. The *M. hieroglyphica* mitogenome provides an important data resource for further studies and contributes to our understanding of the phylogeny of this group.

*Monolepta hieroglyphica* (Motschulsky) belongs to the Galerucinae of Chrysomelidae of Coleoptera (Yu et al. 1996). It is mainly distributed in more than a dozen countries and regions in East Asia and Southeast Asia and is widely distributed in China. It is a high-temperature, drought-type pest and is characterized by its large occurrence area, long damage period, fast reproduction and migratory flight, miscellaneous feeding habits, and a wide range of hosts (Chen et al. 2007). In recent years, the damage to soybean, corn, rice, some vegetables and other crops caused by *M. hieroglyphica* has increased significantly, and its area of occurrence continues to expand (Zhang et al. 2014). There has been considerable research on its prevention and treatment (Tian et al. 2014). In this study, the mitochondrial genome of *M. hieroglyphica* was sequenced and analyzed, and phylogenetic trees were established. The findings are of great significance in providing insights for early warning and management of this pest.

The *M. hieroglyphica* sample was collected from Yinchuan, Ningxia, China (38°54′30″N, 106°32′2″E) in July 2020 and deposited in the insect herbarium of the School of Agriculture, Ningxia University (SANXU; voucher number SBCFYYJ202007-01). The complete mitochondrial genome of *M. hieroglyphica* was sequenced using Illumina HiSeq X Ten. Genes were assembled by MITObim v1.9 (Hahn et al. 2013). Ribosomal RNA (rRNA) and transfer RNA (tRNA) annotations were confirmed and corrected by MITOS online (Bernt et al. 2013).

This mitogenome was a circular DNA molecule, 16,213 bp in length and contained the typical set of 37 genes, including 13 protein-coding genes (PCGs: ATP6, ATP8, COI-III, ND1-6, ND4L and CYTB), two rRNA genes (12S rRNA and 16S rRNA), 22 tRNA genes and a putative CR. The nucleotide composition of the *M. hieroglyphica* mitochondrial genome was 41.04% A, 39.18% T, 11.76% C and 8.01% G; therefore, A + T content was 80.23%. Fourteen genes were located on the H-strand and the others were transcribed on the L-strand. The start codon ATT was shared with COX1, COX2, ND2, ND3, ND5 and ND6; the start codon ATG was shared with COX3, ATP6, ND4, ND4L and CYTB; ATP8 started with codon ATC; and ND1 started with codon TTG. The conservative stop codon TAA was shared with COX1, COX3, ATP6, ATP8, ND2, ND4L and ND6; the stop codon TAG was shared with ND1, ND3 and CYTB; and COX1, ND4 and ND5 terminated with T–. 16 s rRNA and 12 s rRNA were located between tRNA-Leu and the putative CR, separated by tRNA-Val. The 16S rRNA was 1249 bp in length and the 12S rRNA was 744 bp. The mitogenome had a total of 51 bp intergenic spacer sequences and 46 bp overlap sequences, which comprised 8 and 14 regions ranging from 1 to 20 bp and 1 to 8 bp, respectively.

Another 29 Chrysomelidae-Galerucinae species and three outgroups (*Caryopemon giganteus*, *Acanthoscelides obtectus* and *Bruchidius uberatus*) were selected to reconstruct the phylogenetic tree. This was reconstructed using Bayesian 3.2.0 (Ronquist et al. 2012) based on 13 mitochondrial PCGs. The best-fit nucleotide substitution model was selected as ‘GTR + G+I’ using the Akaike Information Criterion (AIC) in jModelTest 0.1.1 (Posada 2008). The phylogenetic analysis showed that the *M. hieroglyphica* mitogenome was clearly clustered with the mitogenomes of the existing three different species of the *Monolepta* genus in a monophyletic manner. It was genetically closest to *Galeruca* sp. and *Apophylia* sp. (Figure 1).

**Figure 1.**
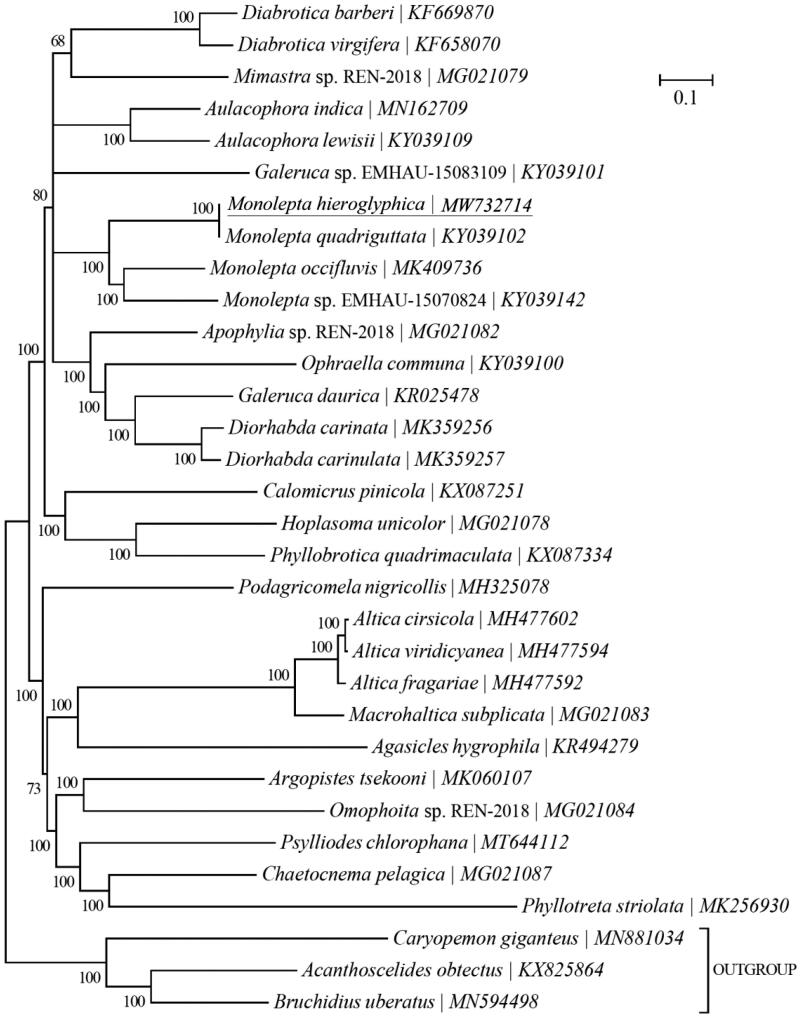
Phylogeny of 29 Chrysomelidae-Galerucinae species based on 13 mitochondrial protein-coding genes reconstructed using Bayesian 3.2.0. The best-fit nucleotide substitution model is ‘GTR + G+I’. The support values are shown next to the nodes. Three Bruchinae species were included as outgroup taxa. Subfamily-level taxonomy was shown for each taxon.

In conclusion, the *M. hieroglyphica* mitogenome sequence will provide a useful data resource for further studies of *M. hieroglyphica* and contributes to understanding of the phylogenetic relationships of the Galerucinae clade.

## Data Availability

The genome sequence data that support the findings of this study are openly available in GenBank of NCBI at [https://www.ncbi.nlm.nih.gov] (https://www.ncbi.nlm.nih.gov/) under the accession no. MW732714. The associated BioProject, SRA, and Bio-Sample numbers are PRJNA713524, SRR13959601 and SAMN18253528, respectively.
