# Correction

**DOI:** 10.1080/23802359.2021.1972998

**Published:** 2021-09-22

Notice of duplicate publication: *The complete mitochondrial genome of Monolepta hieroglyphica (Motschulsky) (Coleoptera: Chrysomelidae)*

Qi He, Xianmei Song, Hongwen Ma and Yanbo Yin, ‘The complete mitochondrial genome of Monolepta hieroglyphica (Motschulsky) (Coleoptera: Chrysomelidae)’*, Mitochondrial DNA Part B* (2021), 6(8), 2363–2365. DOI: https://doi.org/10.1080/23802359.2021.1951138

Please note that this article has been removed from *Mitochondrial DNA Part B* as it is a Duplicate Publication of:

Qi He, Xianmei Song, Hongwen Ma and Yanbo Yin, ‘The complete mitochondrial genome of Monolepta hieroglyphica (Motschulsky) (Coleoptera: Chrysomelidae)’*, Mitochondrial DNA Part B* (2021), 6(7), 2019–2021. DOI: https://doi.org/10.1080/23802359.2021.1926363

